# Plant Responses to Extreme Climatic Events: A Field Test of Resilience Capacity at the Southern Range Edge

**DOI:** 10.1371/journal.pone.0087842

**Published:** 2014-01-28

**Authors:** Asier Herrero, Regino Zamora

**Affiliations:** 1 Department of Ecology, University of Granada, Granada, Andalusia, Spain; 2 Department of Life Sciences, University of Alcalá, Alcalá de Henares, Madrid, Spain; National University of Singapore, Singapore

## Abstract

The expected and already observed increment in frequency of extreme climatic events may result in severe vegetation shifts. However, stabilizing mechanisms promoting community resilience can buffer the lasting impact of extreme events. The present work analyzes the resilience of a Mediterranean mountain ecosystem after an extreme drought in 2005, examining shoot-growth and needle-length resistance and resilience of dominant tree and shrub species (*Pinus sylvestris* vs *Juniperus communis*, and *P. nigra* vs *J. oxycedrus*) in two contrasting altitudinal ranges. Recorded high vegetative-resilience values indicate great tolerance to extreme droughts for the dominant species of pine-juniper woodlands. Observed tolerance could act as a stabilizing mechanism in rear range edges, such as the Mediterranean basin, where extreme events are predicted to be more detrimental and recurrent. However, resistance and resilience components vary across species, sites, and ontogenetic states: adult *Pinus* showed higher growth resistance than did adult *Juniperus*; saplings displayed higher recovery rates than did conspecific adults; and *P. nigra* saplings displayed higher resilience than did *P. sylvestris* saplings where the two species coexist. *P. nigra* and *J. oxycedrus* saplings at high and low elevations, respectively, were the most resilient at all the locations studied. Under recurrent extreme droughts, these species-specific differences in resistance and resilience could promote changes in vegetation structure and composition, even in areas with high tolerance to dry conditions.

## Introduction

Extreme drought and warm events are closely related to growth reductions and mortality of woody species in forest ecosystems across the planet [1]. Recurrent and extreme droughts impact woody species performance differently through species-specific sensitivity, leading to changes in species composition [2], [3], [4], [5], [6], [7]. In this respect, differences in drought sensitivity between functional types, such as trees and shrubs, can alter vegetation structure, shifting from a tree-dominated landscape to a shrub-dominated one [2], [4], [6]. However, stabilizing processes promoting community resilience can palliate and offset the aftermath of extreme events [8]. While resistance can be considered the force of an ecosystem, community or individual to oppose change exerted by an external disturbance [9], resilience is defined as the capacity to restore pre-disturbance structure and function (analogous to ‘engineering resilience’, see [10]). In this context, the analysis of woody species resistance and resilience is particularly crucial under the rising frequency of extreme events [11], [12], [13].

The study of ecosystem responses in terms of resistance and resilience to extreme events can help to forecast ecosystem changes, as future average conditions will be close to current extreme events [14]. At the community level, resistance and resilience after a single extreme event has been related to diversity [15] and resource availability [9]. However, assessments of the consequences of extreme climatic events at the individual and/or population level are limited by a lack of rigorous and testable methods that enable quantifications of plant responses to extreme events under field conditions [8].

The main objective of this study is to analyze the resistance and resilience of a Mediterranean mountain ecosystem to an extreme drought event in 2005, monitoring performance of dominant tree and shrubs species before, during, and afterwards. Boreo-alpine tree *Pinus sylvestris* L. subsp. *nevadensis* Christ and shrub *Juniperus communis* L. are the dominant species along the oromediterranean belt (1800–2000 m a.s.l.), while Mediterranean tree *Pinus nigra* Arnold and shrub *Juniperus oxycedrus* Sibth & Sm are the dominant ones in the supramediterranean belt (1400–1700 m). The species studied were situated close to their southernmost distribution limit, forming natural relict populations in the study area (particularly *P. sylvestris* and *J. communis*; [16]). The impact of extreme climatic events are expected to be more detrimental in populations living at the edge of the distribution range, as those populations are far from that species' optimum conditions. However, observed past persistence in relict populations at rear edges [17] suggest some degree of tolerance to extreme climatic events. Thus, alternatively, the examined rear-edge populations might show an acclimated response to the extreme drought thanks to different stabilizing processes, such as site-specific environmental conditions or stress tolerance capacity linked to local adaptation [8]. Analyses of plant resistance and resilience in rear-edge populations, as in the present work, will help to forecast future shifts in species distributions, as major range contractions are expected in southern ranges [18], [19].

We compare resistance and resilience between species and environmental conditions, considering different ontogenetic states, in order to assess the tree and shrub dominant species response to an extreme drought event. Regarding life form (*Pinus* trees vs. *Juniperus* shrubs), lower resistance can be expected in trees due to stronger stomatal control during drought [20], [7]. With respect to tree species comparison (*P. sylvestris* vs. *P. nigra*), we expect a lower resilience to an extreme drought for *P. sylvestris* due to its boreo-alpine biogeographical origin [21]. Concerning the environmental gradients, higher resilience can be expected for populations located at higher elevations and/or northern exposures than for those at lower elevations and/or southern exposures, because of wetter and cooler conditions in the former. Regarding ontogenetic stage, adults can show alternatively higher resilience to drought than saplings owing to deeper root system, or lower resilience due to higher vulnerability to xylem embolism, greater water use per unit of time [3], and/or slower shoot growth rates [22]. Such comprehensive analysis of tree and shrubs resistance and resilience allows the testing of community tolerance to extreme droughts and the identification of dynamics associated with predicted climatic changes.

In summary, the specific questions addressed in the present study are: 1) Do tree species show lower resilience and resistance than shrubs? 2) Does *P. sylvestris* show lower resilience and resistance than *P. nigra*? 3) Do pine species show lower resilience and resistance at a low elevation and/or southern exposition? 4) Do adults show lower resilience and resistance than saplings?

## Materials and Methods

### Study site and species

The study was conducted at Sierra de Baza Natural Park (SE Spain, 2°51′48″W, 37°22′57″N). All necessary permits for the field studies described herein (which did not involve endangered or protected species) were obtained thanks to Juan Romero, Director of Sierra de Baza Natural Park. The climate is Mediterranean, characterized by cold winters and hot summers, with pronounced summer drought (June-August). Precipitation is concentrated mainly in autumn and spring. The annual and summer rainfall is 495±33 mm and 31±9 mm, respectively (mean ± SE for period 1991–2006; Cortijo Narváez metereological station, 1360 m a.s.l.). The study species are dominant in their altitudinal belt, forming characteristic vegetation types. In the oromediterranean belt (1800–2000 m a.s.l.), while *P. sylvestris* subsp. *nevadensis* is the main tree species, *J. communis* is the main shrub covering the forest understory and open areas. On the other hand, in the supramediterranean belt (1400–1700), *P. nigra* and *J. oxycedrus* are the dominant tree and shrub species, respectively. In 2005 the most extreme drought in the last six decades occurred in Western Europe [23], with climate records in the study area (Cortijo Narváez meteorological station) registering the driest year since 1947.

### Drought index

**Figure 1:**
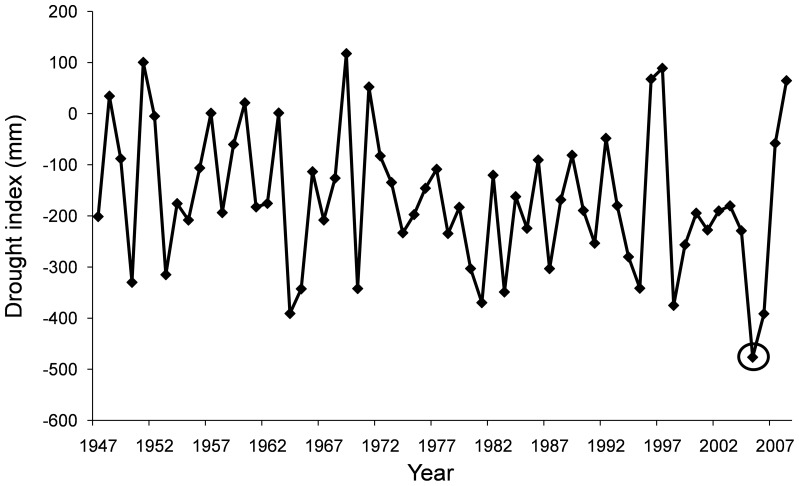
Drought index for 1947–2008 series. 2005, highlighted by a circle, was an extreme drought year.

A drought index (DRI) was calculated for the study site to display the severity of the 2005 extreme drought. The DRI was calculated for the period 1947–2008 using the following formula:
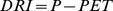
where P is equal to the sum of the precipitation from January to December, and PET equals the sum of estimated potential evapo-transpiration for the same period as a function of monthly mean temperatures and geographical latitude (using Thornthwaite formulation [24]). Monthly total precipitation data was recorded in Cortijo Narváez meteorological station (1360 m a.s.l.; at 900 m to the low altitude plots), very close to the study area. However, monthly mean temperature data was collected from the nearest meteorological station, at Baza village (2°46′24″W, 37°29′23″N), as there are no temperature records in Cortijo Narváez. Temperature data from Baza only cover the period 1990–2009, so data from the CRU TS 2.1 high-resolution gridded data set [25] was used to extend temperature data back to 1947. Linear regressions were performed between local temperature data and the CRU data set, being always significant at *P*<0.05, with R^2^ ranging from 0.41 to 0.89 (approximately 60% of cases showed a R^2^ higher or equal to 0.62). Thereafter, these linear regression equations were used to infer local temperature data from 1947 to 2008. More negative DRI values indicate more severe moisture deficits. DRI data are shown in Figure 1, with 2005 being the lowest value for the period 1947–2008. Thus, we consider 2005 an extreme drought year, since it presented a DRI value located at the lower end of the range of observed values for the studied period [26].

### Sampling design

Different *P. sylvestris* and *P. nigra* populations were monitored in natural relict forests at Sierra de Baza. *P. sylvestris* populations were sampled on north- and south-facing slopes of the same valley (2000 m), while *P. nigra* populations were monitored following an altitudinal gradient: at high (2000 m), medium (1700 m), and low elevations (1500 m). South-facing *P. sylvestris* and high-elevation *P. nigra* populations coincide spatially, forming a mixed forest. *J. communis* and *J. oxycedrus* were sampled at the same north-facing locations of *P. sylvestris* and low-elevation *P. nigra* populations, respectively. For each location, two plots of 1–2 ha each were established, being at least 600 m away from each other. In each plot, large mature adults and non-reproductive saplings were sampled, avoiding individuals with significant herbivory or physical damages. See Table 1 for further information about monitored plots and adult and sapling sizes. All measurements of plant size (height, basal diameter, diameter at breast height, and cover area) were made in late autumn 2008.

**Table 1:** Adults and saplings size in each sampled plot.

				Adults			Saplings		
Species	Altitude	Exposure	Plot	Height (m)^a^	DBH (cm)	Cover area (m^2^)^c^	Height (cm)^a^	Basal diameter (cm)^b^	Cover area (m^2^)^c^
*P. sylvestris*	2065	N	1	9.55±0.69	44.86±2.84	-	112.2±5.85	4.44±0.28	-
*P. sylvestris*	2037	N	2	7.64±0.26	43.35±2.82	-	93.33±8.12	3.68±0.33	-
*P. sylvestris*	2008	S	1	8.89±0.61	43.42±3.92	-	92.63±8.74	5.09±0.64	-
*P. sylvestris*	2067	S	2	7.78±0.36	49.07±3.75	-	110.93±8.33	4.07±0.43	-
*P. nigra*	2008	S	1	9.74±0.67	46.9±4.04	-	111.29±6.55	4.11±0.33	-
*P. nigra*	2067	S	2	9.79±0.62	49.31±2.80	-	99.47±7.51	4.39±0.33	-
*P. nigra*	1753	NE	1	9.6±0.35	34.10±1.20		101.63±6.73	3.3±0.17	
*P. nigra*	1694	NW	2	8.74±0.52	35.86±2.51		103.87±6.10	4.52±0.25	
*P. nigra*	1525	NW	1	8.51±0.6	31.06±2.06	-	99.61±6.18	4.88±0.28	-
*P. nigra*	1544	NE	2	8.69±0.24	33.78±1.03	-	92.63±5.9	4.2±0.17	-
*J. communis*	2065	N	1	-	-	28.57±2.85	-	-	0.31±0.08
*J. communis*	2037	N	2	-	-	14.44±1.18	-	-	0.11±0.02
*J. oxycedrus*	1525	NW	1	1.96±0.08	-	4.29±0.48	0.34±0.02	-	0.06±0.01
*J. oxycedrus*	1544	NE	2	1.9±0.11	-	4.52±0.49	0.44±0.03	-	0.14±0.02

Cover area was calculated measuring maximum and minimum canopy diameters. Values are shown as mean ± standard error. DBH: diameter at breast height.

a Height was not recorded for *J. communis*, as it presents a prostrate growth form.

b Basal diameter was not quantified for the two *Juniperus* species due to measurement difficulties and to the common multi-trunk growth pattern.

c Maximum and minimum canopy diameters were measured to calculate the canopy cover area.

Shoot- and needle-growth resistance and resilience were analyzed in the four dominant tree and shrubs species (*Pinus sylvestris* vs *Juniperus communis*, and *P. nigra* vs *J. oxycedrus*), which showed only one shoot- and needle-growth flush per year in the study area. The existing literature demonstrated that both shoot growth and needle length can be used as indicators of plant responses to water supply, providing a straightforward field sampling measure to analyze short term responses to extreme climatic events in an easy and testable way. For example, shoot growth has been used as an indicator of environmental favorability [27] as well as to measure the impact of drought conditions on plant growth [28], [29], [30], [31]. On the other hand, needle length is also a good indicator of tree responses to water availability [32], [33]. Due to the long retention time of needles in the species considered, branches bore multiple needle cohorts, enabling shoot- and needle-growth changes to be easily compared.

#### Trees

For tree species, 10 representative mature trees and 15 saplings of similar size were recorded haphazardly in each plot. Height and DBH (diameter at breast height) in adults, and height, basal diameter, and age in saplings were recorded (see Table 1). Adult height was measured using a Vertex IV hypsometer (Haglöf, Sweden). Sapling age was estimated by counting the number of annual bud scars or whorls [34], [35], [36] as the two pine species showed one flush per year in the study area. Longitudinal shoot growth in adults was measured in 10 branches per tree, five facing north and five south. All the branches were tagged, measuring the same branches in the different samplings. Measured branches belonged to medium or low tree crown. Values from the ten branches were averaged to obtain a unique value per individual for each year. In saplings, shoot growth was measured in the leader shoot. Shoot growth of each year was identified using annual whorls and yearly bud scars from 2003 to 2008. Needle length was measured in three needles per shoot-growth cohort, which were randomly recorded. Shoot growth and needle length were measured from winter 2006 to late autumn 2008.

#### Shrubs

In each plot, 20 adults (10 males and 10 females) and 20 saplings of similar size were haphazardly recorded. All adults were pooled due to the absence of significant differences between sexes in the recorded variables. Annual longitudinal shoot growth was measured in 10 and 5 branches for adults and saplings, respectively. Values from the measured branches were averaged to obtain a unique value per individual for each year. Measurements were made from the 2004 to 2008 cohort based on differences in color and diameter showed by the different cohorts. Needle length was also measured in three needles of each shoot-growth cohort. Shoot growth and needle length were measured from winter 2006 to late autumn 2008.

### Resistance and resilience components

**Figure 2:**
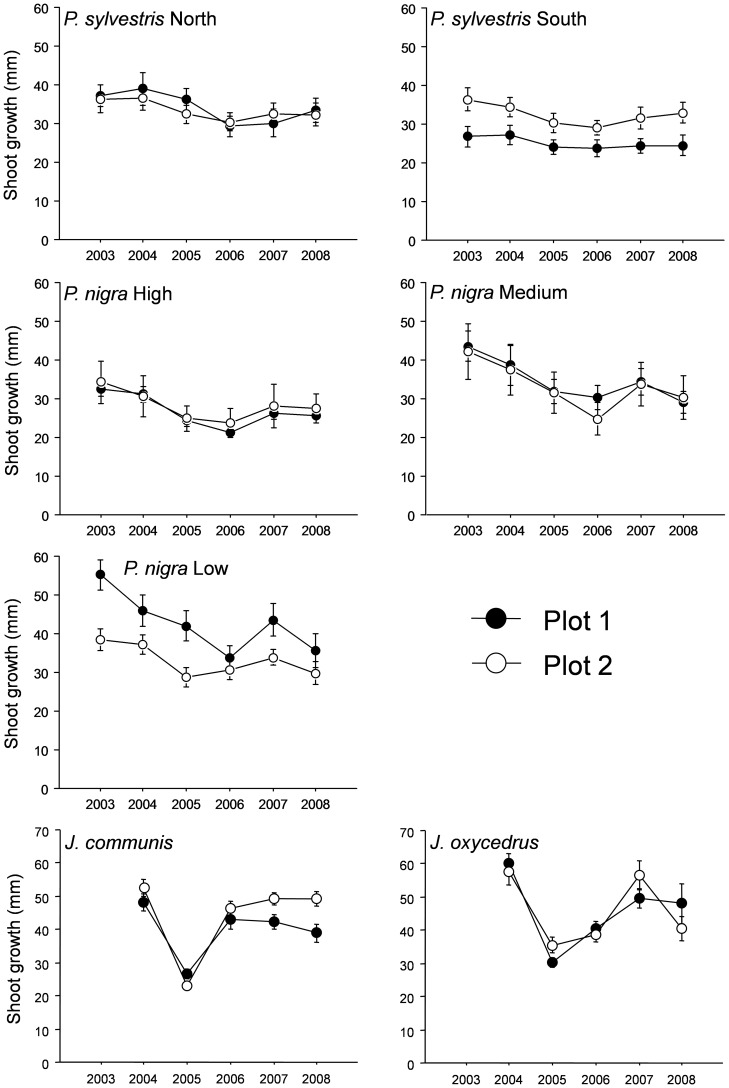
Shoot growth for the period 2003–2008 for adults of the four species at sampled locations. Data for a *Pinus sylvestris* with southern and northern exposure, for *P. nigra* at high (2000 m), medium (1700 m) and low elevation (1500 m), and for *Juniperus communis* and *J. oxycedrus* are shown.

To analyze resistance and resilience to 2005 extreme drought in shoot growth and needle length of considered species, we calculated resistance, recovery, resilience, and relative resilience for both variables following the procedure of Lloret and others [37]. Resistance, the inverse of the performance reduction during the extreme drought, was calculated as the ratio between performance during and before drought. Recovery, the ability to recover relative to the performance reduction undergone during drought, was calculated as the ratio between performance after and during the extreme drought. Resilience, the capacity to return to pre-drought performance levels, was calculated as the ratio between the performance after and before drought. Relative resilience is the resilience weighted by the performance reduction during drought and was calculated using the following formula:
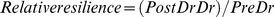
where *PreDr*, *Dr* and *PostDr* indicate performance before, during, and after drought, respectively. Performance before and after drought were calculated as the average over a two-year period, and performance during drought as the values for the year 2005. However, we made some modifications taking into account the shoot-growth patterns of the species studied. For the shoot growth of pine species, 2003 and 2004 corresponded to pre-drought values, 2005 and 2006 to during-drought values, and 2007 and 2008 to post-drought values. In 2005, extreme drought affected 2005 and 2006 pine shoot cohorts, as the conditions during bud formation can affect the following year's shoot growth [38], [39]. For shoot growth of shrub species, 2004 corresponded to pre-drought values, 2005 to during-drought values, and 2007 and 2008 to post-drought values. Pre-drought values included only 2004, as the identification of 2003 shoot cohort was not possible when the study began (winter 2006). In contrast to *Pinus*, *Juniperus* presented an indeterminate shoot growth, with only 2005 shoot cohort being affected by the extreme drought (see Fig. 1 and 2). Although only 2007 and 2008 were considered for post-drought values, the inclusion of 2006 values did not change the results. Finally, for needle growth, drought values include only the 2005 cohort for both pines and shrubs, as needle length appeared to respond to the dry conditions of the current season (Fig. S1 and S2; see also [38]).

### Data analysis

Shoot-growth and needle-length resistance, recovery, resilience, and relative resilience were analyzed in a search for differences between species and locations (exposure and altitude) considering two different ontogenetic states (large adults/non-reproductive saplings). Our ‘experimental unit’ was the individual tree or shrub, for which shoot-growth and needle-length values were averaged. Afterwards, different resistance and resilience components were calculated as explained above. Three species comparisons were performed: 1) *P. sylvestris* vs. *J. communis* with a northern exposure at a high elevation; 2) *P. sylvestris* vs. *P. nigra* with a southern exposure at a high elevation; and 3) *J. oxycedrus* vs. *P. nigra* at a low elevation. For locations, two comparisons were made: 1) between northern and southern exposures for *P. sylvestris*; and 2) between high, medium, and low elevations for *P. nigra*. Differences between species and locations were analyzed using General Linear Mixed Models (GLMM), with species (or location), ontogenetic state and their interaction as fixed factors, and plot as a random factor. Shoot-growth or needle-length resistance, recovery, resilience or relative resilience was used as the dependent variable in each case. *Post hoc* comparisons between groups were performed using Tukey's HSD test. All the analyses were performed using JMP 7.0 (SAS Institute Inc.). All results throughout this paper are given as mean ± standard error.

## Results

### Shoot growth

**Figure 3:**
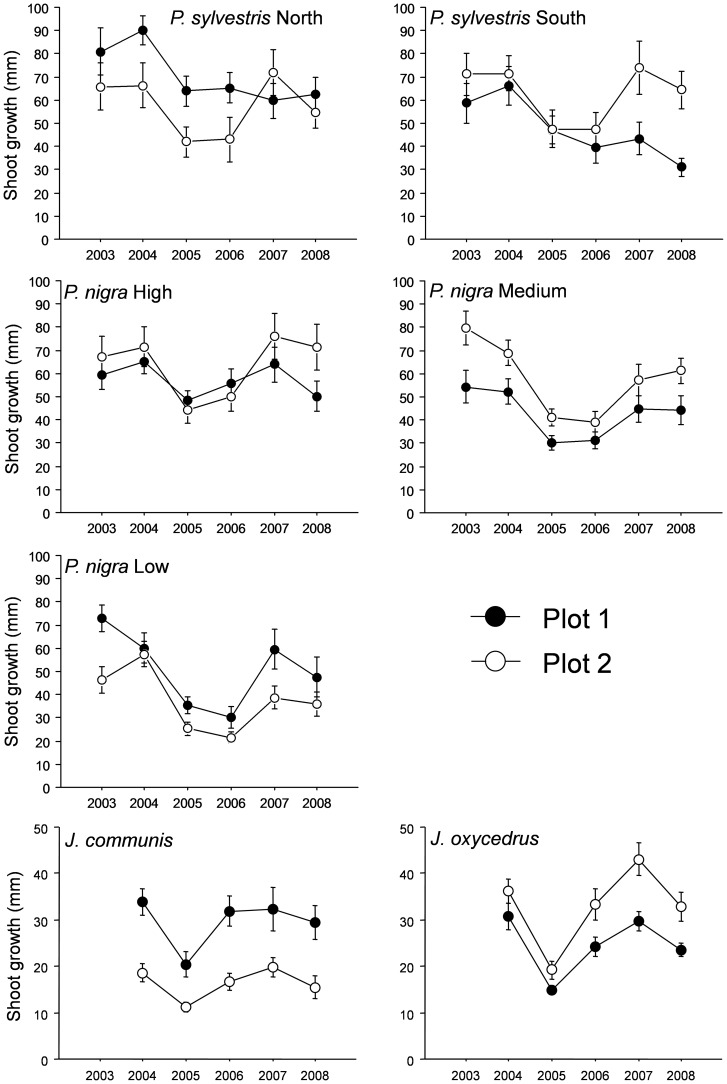
Shoot growth for the period 2003–2008 for saplings of the four species at sampled locations. Data for a *Pinus sylvestris* with southern and northern exposure, for *P. nigra* at high (2000 m), medium (1700 m) and low elevation (1500 m), and for *Juniperus communis* and *J. oxycedrus* are shown.

Figure 2 and 3 showed shoot growth for the period 2003–2008 (2004–2008 for *Juniperus*) for adults and saplings of the considered four species at sampled locations.

#### *P. sylvestris* vs. *J. communis*

**Figure 4:**
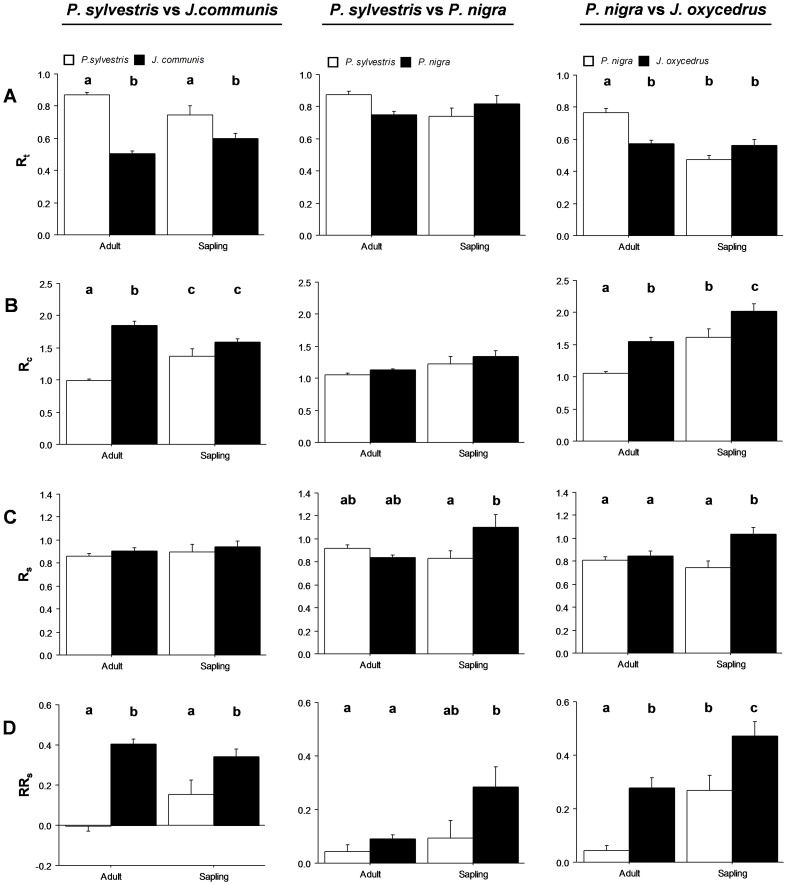
Differences in shoot-growth resistance (A), recovery (B), resilience (C) and relative resilience (D) between species and ontogenetic states (adults/saplings). Three comparisons are shown: *P. sylvestris* vs. *J. communis* with a northern exposure at high elevation; *P. sylvestris* vs. *P. nigra* with a southern exposure at high elevation; and *J. oxycedrus* vs. *P. nigra* at low elevation. Different letters above bars indicate significant *post hoc* differences between groups. Bars indicate the standard errors of calculated means.

*P. sylvestris* presented significantly higher resistance but lower relative resilience than did *J. communis* for adults as well as saplings (Fig. 4A, 4D; Table 2). Adult *P. sylvestris* showed slightly negative relative resilience, underlining the incomplete recovery in shoot growth for this case (Fig. 4D; Table 2). On the other hand, *J. communis* adults presented significantly higher recovery than did *P. sylvestris* adults (Fig. 4B; Table 2).

**Table 2:** Summary of GLMM analysis for shoot-growth resistance (R_t_), recovery (R_c_), resilience (R_s_), and relative resilience (RR_s_) for species and location comparisons.

	R_t_		R_c_		R_s_		RR_s_	
	F	*P*	F	*P*	F	*P*	F	*P*
*P. sylvestris* vs. *J. communis*								
Species	53.452	**<0.0001**	52.534	**<0.0001**	0.820	0.367	51.972	**<0.0001**
Ontogenetic state (Ont)	0.159	0.690	0.464	0.4968	0.593	0.443	1.486	0.2251
Species x Ont	10.132	**0.0018**	18.854	**<0.0001**	0.0001	0.991	7.289	**0.0079**
*P. sylvestris* vs. *P. nigra*								
Species	0.196	0.658	1.145	0.2874	1.637	0.2039	4.077	**0.0463**
Ontogenetic state (Ont)	0.501	0.481	4.491	**0.0367**	1.343	0.2494	4.299	**0.0408**
Species x Ont	4.783	**0.031**	0.069	0.7928	5.277	**0.0238**	1.556	0.2153
*P. nigra* vs. *J. oxycedrus*								
Species	2.915	0.0902	15.614	**0.0001**	8.719	**0.0038**	18.613	**<0.0001**
Ontogenetic state (Ont)	21.939	**<0.0001**	20.553	**<0.0001**	1.148	0.286	17.438	**<0.0001**
Sp x Ont	18.962	**<0.0001**	0.112	0.738	5.194	**0.0244**	0.098	0.754
*P. sylvestris*: Exposure								
Exposure	0.0001	0.992	0.206	0.651	0.014	0.905	0.013	0.907
Ontogenetic state (Ont)	6.796	**0.010**	7.623	**0.006**	0.201	0.655	3.809	0.053
Exposure x Ont	0.013	0.910	1.085	0.300	1.221	0.272	1.099	0.297
*P. nigra*: Altitude								
Altitude	10.216	**<0.0001**	0.633	0.532	4.258	**0.016**	0.244	0.784
Ontogenetic state (Ont)	16.649	**<0.0001**	27.785	**<0.0001**	2.359	0.127	25.598	**<0.0001**
Altitude x Ont	12.081	**<0.0001**	1.729	0.181	2.947	0.055	0.044	0.956

Species comparisons comprise *P. sylvestris* vs. *J. communis*, *P. sylvestris* vs. *P. nigra*, and *P. nigra* vs. *J. oxycedrus*. Location comparisons comprise exposure and altitude differences for *P. sylvestris* and *P. nigra*, respectively.

#### *P. sylvestris* vs. *P. nigra*

Saplings of both species displayed significantly higher recovery and relative resilience than conspecific adults, relative resilience also being significantly higher in *P. nigra* (Fig. 4B, 4D; Table 2). Finally, *P. nigra* saplings showed the greatest resilience values (Fig. 4C; Table 2).

#### *P. nigra* vs. *J. oxycedrus*

*J. oxycedrus* showed significantly higher recovery and relative resilience than *P. nigra*, values being significantly higher in saplings (Fig. 4B, 4D; Table 2). *P. nigra* adults showed significantly higher resistance than did conspecific saplings and *J. oxycedrus*, while *J. oxycedrus* saplings showed significantly higher resilience than did conspecific adults and *P. nigra* (Fig. 4A, 4C; Table 2).

#### *P. sylvestris*: Exposure

**Figure 5:**
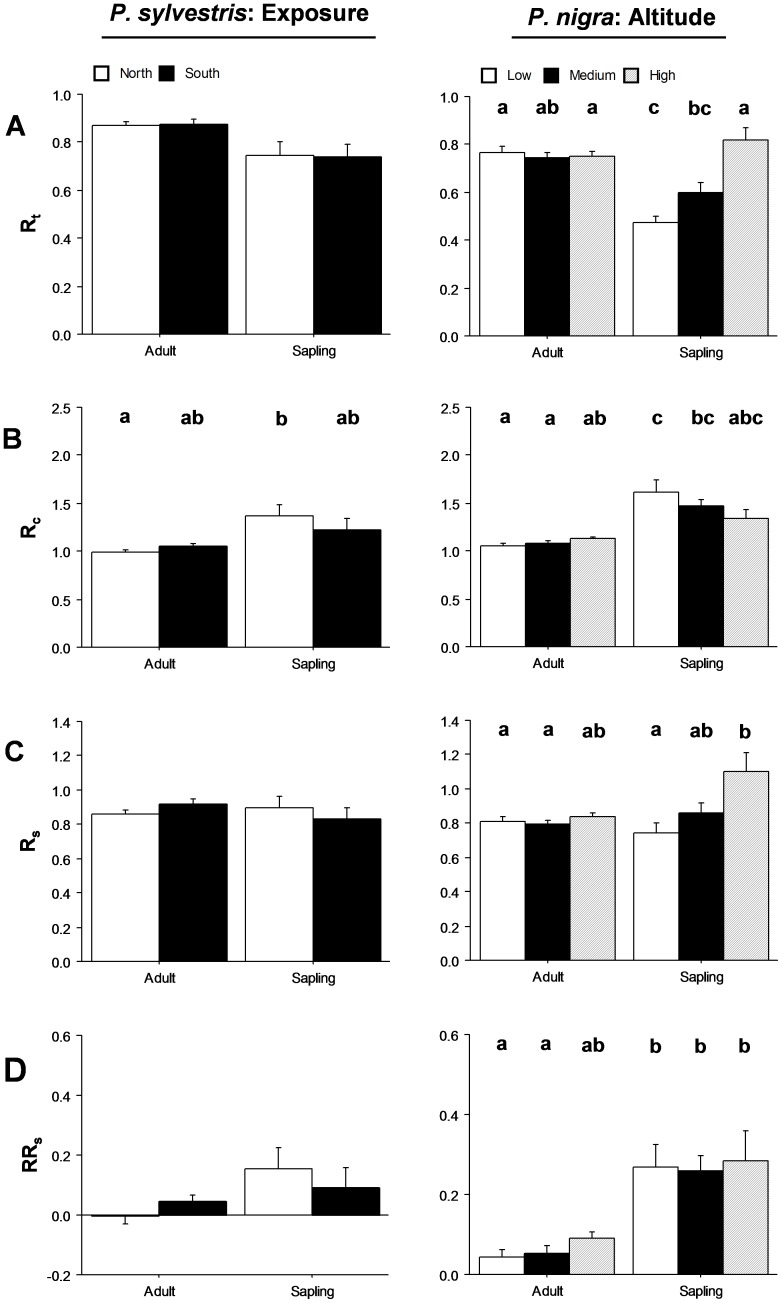
Differences in shoot-growth resistance (A), recovery (B), resilience (C), and relative resilience (D) between locations and ontogenetic states (adults/saplings). Two comparisons are shown: between northern and southern exposure for *P. sylvestris*; and between high (2000 m), medium (1700 m), and low (1500 m) elevations for *P. nigra*. Different letters above bars indicate significant *post hoc* differences between groups. Bars indicate the standard errors of calculated means.

No significant differences were found between exposures in any resistance and resilience components (Fig. 5; Table 2). While adults showed significantly higher resistance than saplings, saplings showed significantly higher recovery (Fig. 5A, 5B; Table 2).

#### *P. nigra*: Altitude

Adults showed significantly lower recovery and relative resilience values than saplings (Fig. 5B, 5D; Table 2). Differences in altitude clearly appeared between saplings, with resistance and resilience being significantly stronger at the high altitude than at the low one (Fig. 5A, 5C; Table 2).

### Needle length

Figure S1 and S2 showed needle length for the period 2003–2008 (2004–2008 for *Juniperus*) for adults and saplings of the considered four species at sampled locations.

#### *P. sylvestris* vs. *J. communis*

*J. communis* showed significantly higher resistance and resilience than *P. sylvestris* for both adults and saplings (Fig. S3A, S3C; Table 3). Furthermore, adults of *J. communis* displayed significantly higher recovery than did conspecific saplings and *P. sylvestris* (Fig. S3B; Table 3).

**Table 3:** Summary of GLMM analysis for needle-length resistance (R_t_), recovery (R_c_), resilience (R_s_), and relative resilience (RR_s_) for species and location comparisons.

	R_t_		R_c_		R_s_		RR_s_	
	F	*P*	F	*P*	F	*P*	F	*P*
*P. sylvestris* vs. *J. communis*								
Species	21.777	**<0.0001**	1.947	0.165	32.224	**<0.0001**	0.020	0.886
Ontogenetic state (Ont)	1.340	0.249	4.297	**0.0402**	0.159	0.690	1.527	0.219
Species x Ont	0.107	0.743	4.723	**0.0316**	4.497	**0.0359**	2.829	0.095
*P. sylvestris* vs. *P. nigra*								
Species	0.268	0.606	9.253	**0.0031**	8.606	**0.0043**	4.885	**0.0298**
Ontogenetic state (Ont)	2.888	0.093	10.187	**0.0020**	2.266	0.136	10.819	**0.0015**
Species x Ont	0.449	0.505	3.320	0.072	1.055	0.307	0.945	0.333
*P. nigra* vs. *J. oxycedrus*								
Species	63.698	**<0.0001**	74.103	**<0.0001**	4.1915	**0.0427**	57.762	**<0.0001**
Ontogenetic state (Ont)	23.592	**<0.0001**	49.452	**<0.0001**	0.075	0.7842	32.525	**<0.0001**
Sp x Ont	4.296	**0.0403**	25.462	**<0.0001**	0.097	0.7553	8.393	**0.0045**
*P. sylvestris*: Exposure								
Exposure	1.165	0.283	6.180	**0.0147**	4.334	**0.0401**	1.810	0.182
Ontogenetic state (Ont)	1.472	0.228	6.030	**0.0159**	1.490	0.225	5.816	**0.0179**
Exposure x Ont	0.007	0.932	0.831	0.364	0.287	0.593	0.231	0.632
*P. nigra*: Altitude								
Altitude	17.210	**<0.0001**	9.760	**0.0001**	1.894	0.154	4.525	**0.0125**
Ontogenetic state (Ont)	40.032	**<0.0001**	55.928	**<0.0001**	0.418	0.519	54.036	**<0.0001**
Altitude x Ont	2.996	0.0533	2.850	0.0613	0.935	0.395	0.067	0.934

Species comparisons comprise *P. sylvestris* vs. *J. communis*, *P. sylvestris* vs. *P. nigra* and *P. nigra* vs. *J. oxycedrus*. Location comparisons comprise exposure and altitude differences for *P. sylvestris* and *P. nigra*, respectively.

#### *P. sylvestris* vs. *P. nigra*

*P. nigra* displayed significantly higher recovery and relative resilience than *P. sylvestris*, values being significantly higher in saplings (Fig. S3B, S3D; Table 3). *P. nigra* also showed significantly higher resilience than did *P. sylvestris* (Fig. S3C; Table 3).

#### *P. nigra* vs. *J. oxycedrus*

*J. oxycedrus* showed higher resistance but lower recovery and relative resilience than did *P. nigra*, with saplings showing lower resistance but higher recovery and relative resilience (Fig. S3; Table 3). Finally, *J. oxycedrus* showed significantly higher resilience than did *P. nigra* (Fig. S3C; Table 3).

#### *P. sylvestris*: Exposure

*P. sylvestris* having a northern exposure showed significantly higher recovery and resilience than having a southern exposure, with saplings showing significant higher recovery (Fig. S4B, S4C; Table 3). Saplings presented also significantly higher relative resilience than did adults (Fig. S4D, Table 3).

#### *P. nigra*: Altitude

*P. nigra* trees showed significant differences in altitude for resistance, recovery, and relative resilience, especially for saplings (Fig. S4, Table 3). For resistance, the highest values were for high-elevation individuals and the lowest for low-elevation ones, showing the opposite pattern in the case of recovery and relative resilience (Fig. S4).

## Discussion

In this study, we empirically apply the concepts of resistance and resilience to patterns of tree and shrub growth, using shoot-length and needle-length as indicators of plant responses to an extreme drought event. The 2005 drought was the most extreme drought in the study area in the last six decades, even triggering pine sapling mortality in the nearby Sierra Nevada [40]. Our empirical results indicate that *Pinus* and *Juniperus* species at their southern distribution edge present great tolerance to an extreme drought event, as demonstrated by the high vegetative (shoot and needle growth) resilience values recorded across species, sites, and ontogenetic states. In fact, resilience values were in general higher than 0.8 which indicate that post-drought values were close to pre-drought ones (R_t_ = 1 indicate identical growth values before and after drought). Thus, the impact of the 2005 extreme drought after three years was rather low, supporting our hypothesis that dominant species of Mediterranean pine-juniper woodlands presents high tolerance and resilience to extreme droughts at their southern distribution edge. Although we cannot compare resilience capacity of southern populations with northern ones, which is beyond the scope of this study, our results are of special relevance under the climate change scenario, since strong distributional shifts and local extinctions are expected at the southern range edge associated with increasing aridity conditions [18], [19].

Observed tolerance ability at the study area could be related to plant adaptation to Mediterranean dry conditions. In fact, high genetic differentiation of southern *P. sylvestris* and *P. nigra* populations [41], [42] suggest high adaptation to the local environment. For instance, *P. sylvestris* population at the study area showed lower vulnerability to embolism than did other Northern European populations [43]. In addition, in an experimental study, Mediterranean *P. sylvestris* provenance showed higher emergence and survival than more northern provenance under different precipitation regimes [44]. Overall, the study species might present specific resilience component values above a hypothetic mortality threshold [37], as no die-back symptoms were detected. It is important to note that no mortality was observed in the study area associated with the 2005 extreme drought. Thus, dominance and maintenance of pine-juniper woodlands in Mediterranean mountains are fostered by the remarkable survival ability and longevity of mature individuals (persistence, *sensu* [45]) as well as high tolerance to extreme droughts of adults and saplings.

Despite that the overall high resilience, resistance and resilience components varied across species and ontogenetic states. Adults of both *Juniperus* species showed lower growth resistance (greater reduction of growth) than did *Pinus* adults. We expected the opposite pattern, as *Juniperus* present an anisohydric regulation, allowing higher stomatal conductance and thus higher photosynthetic uptake to be sustained under dry conditions than in isohydric *Pinus* [20], [7]. However, the deeper root system of trees presumably provides them access to deeper groundwater, thereby boosting stomatal conductance during the 2005 extreme drought [46]. But *Juniperus* species displayed higher relative resilience than did *Pinus* species, both for saplings and for adults, revealing the capacity of *Juniperus* to recover from heavier growth reductions than *Pinus* after an extreme drought event. Of special importance are the high resilience values registered by *J. oxycedrus* saplings at the low elevation, in comparison with coexisting *P. nigra* saplings. Higher drought-induced mortality for *Pinus* species in comparison with *J. monosperma* in the western USA [2], [6], [47] suggests less mortality risk for *Juniperus* species. Thus, differences in growth resilience between *P. nigra* and *J. oxycedrus* at sapling stage, as well as mortality risk for adults, could encourage a shift towards a shrub dominated forest at low elevations, as has been reported in other pine-juniper woodlands [2], [6], [47]. Overall, the extreme drought impact was stronger at the low altitude, as recorded in other studies [2], [48], [49].

Similarly, higher resilience of *P. nigra* saplings than *P. sylvestris* ones, may play an important role under a scenario of recurrent extreme droughts. Several studies indicate higher vulnerability to drought for *P. sylvestris* than for *P. nigra* over ontogeny in locations where the two species coexist [50], [51], [52], [40]. In fact, in the last few years, drought-induced growth declines and mortality events have been recorded in many southern *P. sylvestris* populations [4], [5], [31], [53], [40]. Biotic factors, such pests or browsing, can exacerbate drought vulnerability, inflicting severe damage [54], [55]. In the study area, higher ungulate preference for *P. sylvestris* over *P. nigra* reinforced their climatic responses at the treeline, aggravating drought vulnerability of *P. sylvestris* [55]. Therefore, the higher resilience of *P. nigra* saplings, coupled with its lower vulnerability to drought and browsing, could favor a change in dominance toward this Mediterranean species at high elevations.

In general, both *Pinus* and *Juniper* saplings showed higher recovery than did adults for all the exposures and elevations considered. This recovery capacity might be due to the observed higher shoot-growth rate in saplings than in adults [22], promoting growth recovery after the extreme drought. In addition, *P. nigra* and *J. oxycedrus* saplings at high and low elevations, respectively, were the most resilient in terms of shoot growth. In fact, they were the only cases where shoot-growth resilience reached values higher than one, indicating greater growth values after drought than before drought.

Our study provided a new perspective on the analysis of vegetation responses to climatic events at the individual and population level. Differences in resistance and resilience between dominant tree and shrub species, as observed in this study, can heavily influence vegetation dynamics. Under recurrent extreme droughts, and progressively warmer and drier conditions, such differences can promote changes in both structure and composition of vegetation along a gradient of environmental conditions, even in areas with high tolerance to dry conditions, such as the southern range edge. Our results are useful for forecasting plant responses and distributional shifts under a climate-change scenario, especially at species distribution limits such as the Mediterranean basin, where extreme events are predicted to be more detrimental and recurrent [56], [57]. More interestingly, the great tolerance and/or higher recovery capacity to extreme droughts of both *Pinus* and *Juniper* species should be taken in account when species responses are modeled to future climatic conditions, as models predict sharp decreases in plant diversity and performance in Mediterranean mountains [19], [57], [58]. Our empirical results also indicated that, for an accurate evaluation of the resistance/resilience ability of current vegetation under a climate-change scenario, a realistic modeling approach requires empirical data to analyze plant responses to extreme events at the individual and population levels, considering both different environmental conditions and ontogenetic states (adults vs. saplings).

## Supporting Information

Figure S1 Needle length for the period 2003–2008 for adults of the four species at sampled locations. Data for a *Pinus sylvestris* with southern and northern exposure, for *P. nigra* at high (2000 m), medium (1700 m) and low elevation (1500 m), and for *Juniperus communis* and *J. oxycedrus* are shown.
(TIF)

Figure S2 Needle length for the period 2003–2008 for saplings of the four species at sampled locations. Data for a *Pinus sylvestris* with southern and northern exposure, for *P. nigra* at high (2000 m), medium (1700 m) and low elevation (1500 m), and for *Juniperus communis* and *J. oxycedrus* are shown.
(TIF)

Figure S3 Differences in needle-length resistance (A), recovery (B), resilience (C) and relative resilience (D) between species and ontogenetic states (adults/saplings). Three comparisons are shown: *P. sylvestris* vs. *J. communis* with a northern exposure at high elevation; *P. sylvestris* vs. *P. nigra* with a southern exposure at high elevation; and *J. oxycedrus* vs. *P. nigra* at low elevation. Different letters above bars indicate significant *post hoc* differences between groups. Bars indicate the standard errors of calculated means.
(TIF)

Figure S4 Differences in needle-length resistance (A), recovery (B), resilience (C), and relative resilience (D) between locations and ontogenetic states (adults/saplings). Two comparisons are shown: between northern and southern exposure for *P. sylvestris*; and between high (2000 m), medium (1700 m), and low (1500 m) elevations for *P. nigra*. Different letters above bars indicate significant *post hoc* differences between groups. Bars indicate the standard errors of calculated means.
(TIF)
