# Supplementary figures and images for: Plant Responses to Extreme Climatic Events: A Field Test of Resilience Capacity at the Southern Range Edge
Source: PLoS One. 2014 Jan 28;9(1):e87842. doi: 10.1371/journal.pone.0087842 (PMC3905046; doi:10.1371/journal.pone.0087842)

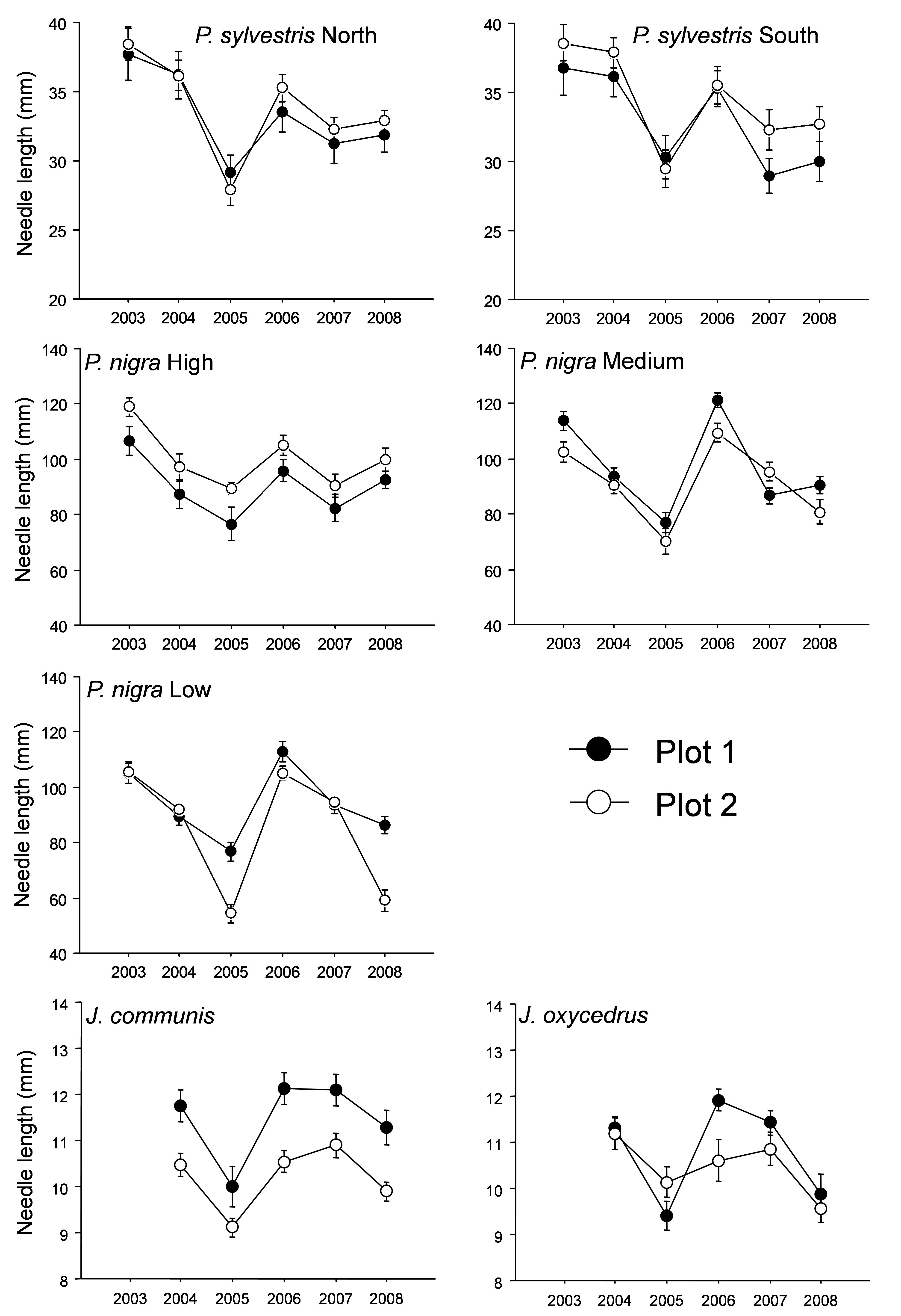

Supplement: Figure S1 — Needle length for the period 2003–2008 for adults of the four species at sampled locations. Data for a Pinus sylvestris with southern and northern exposure, for P. nigra at high (2000 m), medium (1700 m) and low elevation (1500 m), and for Juniperus communis and J. oxycedrus are shown. (TIF) [file pone.0087842.s001.tif]

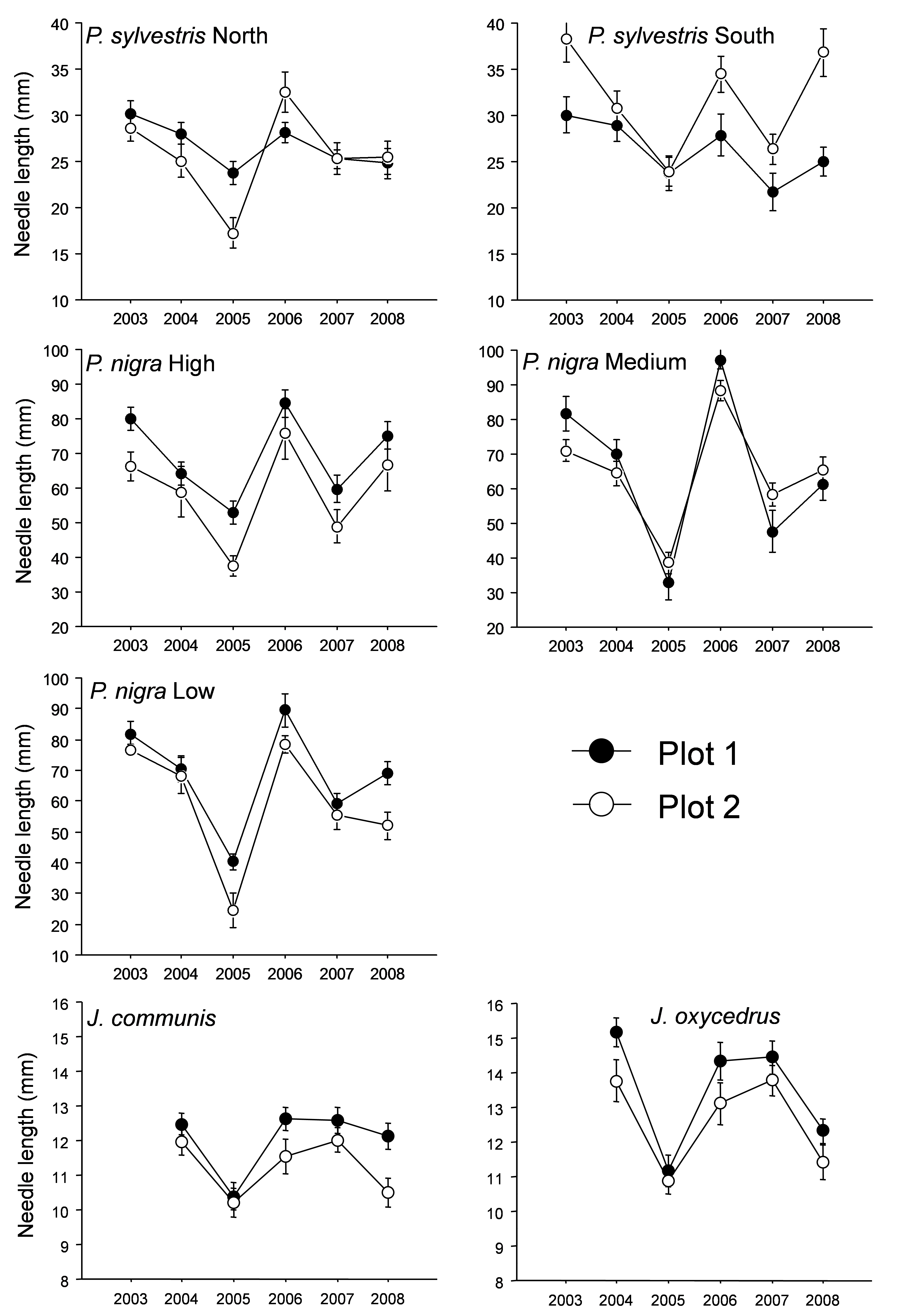

Supplement: Figure S2 — Needle length for the period 2003–2008 for saplings of the four species at sampled locations. Data for a Pinus sylvestris with southern and northern exposure, for P. nigra at high (2000 m), medium (1700 m) and low elevation (1500 m), and for Juniperus communis and J. oxycedrus are shown. (TIF) [file pone.0087842.s002.tif]

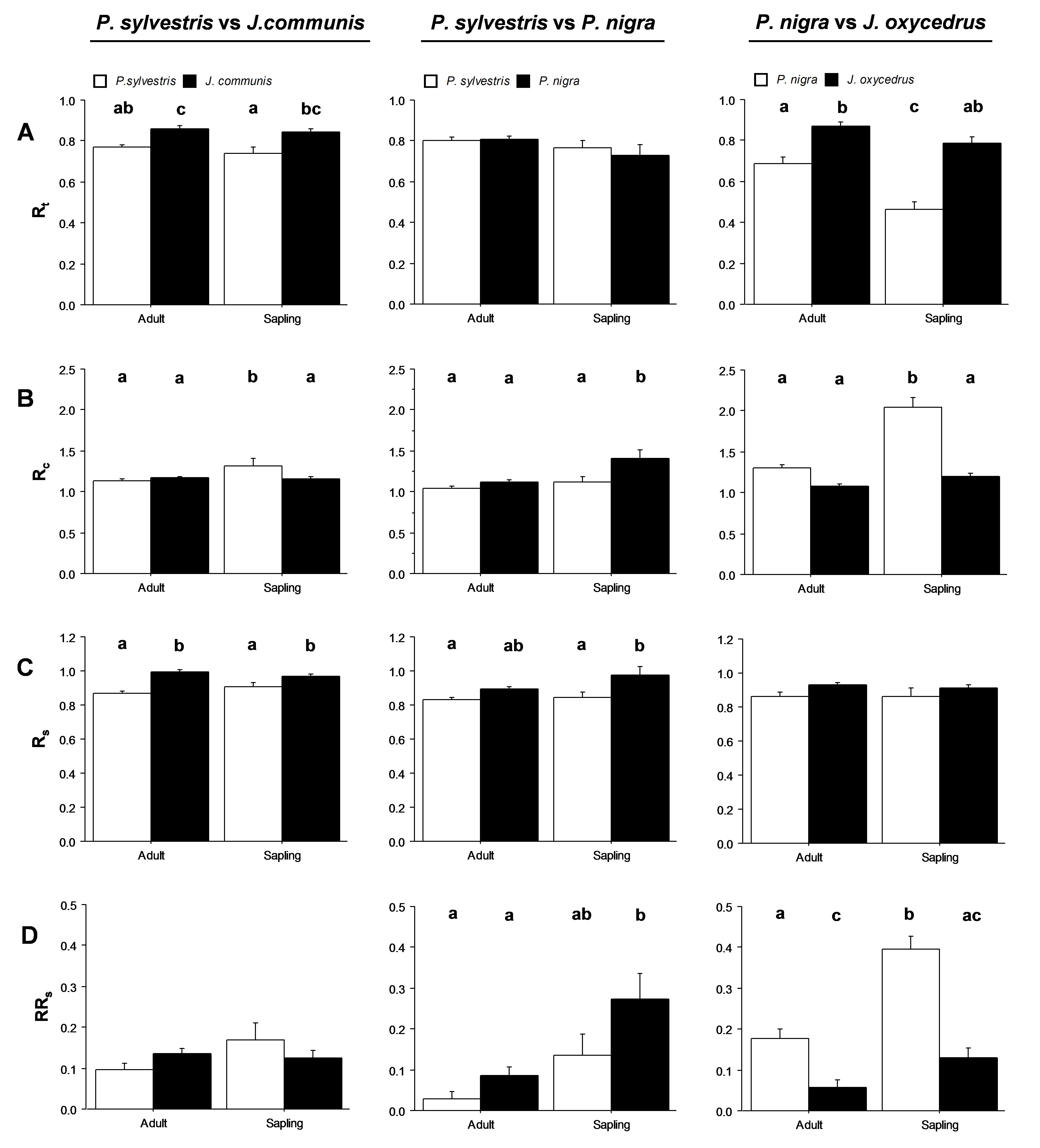

Supplement: Figure S3 — Differences in needle-length resistance (A), recovery (B), resilience (C) and relative resilience (D) between species and ontogenetic states (adults/saplings). Three comparisons are shown: P. sylvestris vs. J. communis with a northern exposure at high elevation; P. sylvestris vs. P. nigra with a southern exposure at high elevation; and J. oxycedrus vs. P. nigra at low elevation. Different letters above bars indicate significant post hoc differences between groups. Bars indicate the standard errors of calculated means. (TIF) [file pone.0087842.s003.tif]

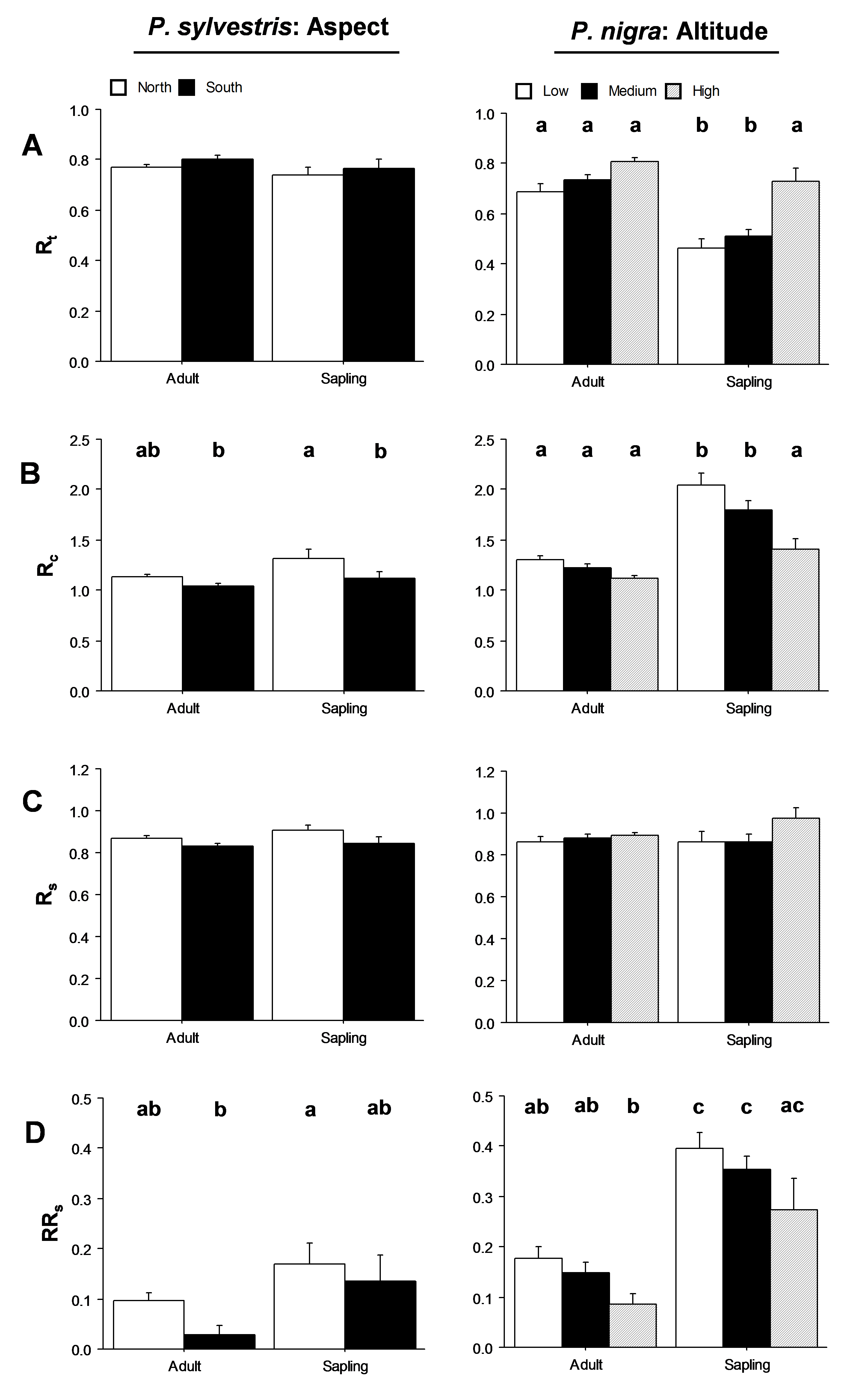

Supplement: Figure S4 — Differences in needle-length resistance (A), recovery (B), resilience (C), and relative resilience (D) between locations and ontogenetic states (adults/saplings). Two comparisons are shown: between northern and southern exposure for P. sylvestris; and between high (2000 m), medium (1700 m), and low (1500 m) elevations for P. nigra. Different letters above bars indicate significant post hoc differences between groups. Bars indicate the standard errors of calculated means. (TIF) [file pone.0087842.s004.tif]
